# Amyotrophic Lateral Sclerosis and Occupational Exposures: A Systematic Literature Review and Meta-Analyses

**DOI:** 10.3390/ijerph15112371

**Published:** 2018-10-26

**Authors:** Lars-Gunnar Gunnarsson, Lennart Bodin

**Affiliations:** 1 School of Medical Sciences, Örebro University, SE 701 82 Örebro, Sweden; 2 Department of Occupational and Environmental Medicine, Örebro University, SE 701 82 Örebro, Sweden; 3 Department of Statistics, Örebro University, SE 701 82 Örebro, Sweden; lennart.bodin@oru.se; 4 Institute of Environmental Medicine, Karolinska Institute, SE 177 77 Stockholm, Sweden

**Keywords:** epidemiology, metals, chemicals, physical activity, electromagnetic fields

## Abstract

*Objectives:* We conducted a systematic literature review to identify studies fulfilling good scientific epidemiological standards for use in meta-analyses of occupational risk factors for amyotrophic lateral sclerosis (ALS). *Methods*: We identified 79 original publications on associations between work and ALS. The MOOSE (Meta-analysis Of Observational Studies in Epidemiology) and GRADE (Grading of Recommendations, Assessment, Development and Evaluations) guidelines were used to ensure high scientific quality, and reliable protocols were applied to classify the articles. Thirty-seven articles fulfilled good scientific standards, while 42 were methodologically deficient and thus were excluded from our meta-analyses. *Results*: The weighted relative risks for the various occupational exposures were respectively; 1.29 (95% confidence interval (CI): 0.97–1.72; six articles) for heavy physical work, 3.98 (95% CI: 2.04–7.77; three articles) for professional sports, 1.45 (95% CI: 1.07–1.96; six articles) for metals, 1.19 (95% CI: 1.07–1.33; 10 articles) for chemicals, 1.18 (95% CI: 1.07–1.31; 16 articles) for electromagnetic fields or working with electricity, and 1.18 (95% CI: 1.05–1.34; four articles) for working as a nurse or physician. *Conclusions*: Meta-analyses based only on epidemiologic publications of good scientific quality show that the risk of ALS is statistically significantly elevated for occupational exposures to excessive physical work, chemicals (especially pesticides), metals (especially lead), and possibly also to electromagnetic fields and health care work. These results are not explained by publication bias.

## 1. Introduction

The average onset of amyotrophic lateral sclerosis (ALS) is between 58 and 60 years of age and the incidence is around two per 100,000 persons [1]. The disease is characterized by an adult-onset progressive degeneration of the motor neurons. The resulting paresis begins focally, spreads contiguously and causes death from respiratory failure when breathing muscles become involved [2].

Fifty years ago, an observational study indicated that heavy work load was associated with ALS [3]. Since then many epidemiological studies have been performed on diverse risk factors. Eight systematic literature reviews with meta-analyses have been published regarding associations between ALS and occupational exposures. Exposure to electromagnetic fields was the focus of four studies [4,5,6,7], out of which two were meta-analyses [5,6]. They concluded that there might be a moderate association with ALS but the results were hampered by the heterogeneity between the included studies. Two meta-analyses showed that exposure to pesticides resulted in an almost doubled risk for ALS [8,9]. Another meta-analysis based on nine case-control studies showed that exposure to lead also involved an almost doubled risk for ALS [10]. The last meta-analysis focused on physical activity as a potential risk factor, but the results were inconclusive [11].

In two studies, attempts were made only to include publications of acceptable scientific quality [5,11]. All the other meta-analyses included all relevant publications, irrespective of whether the studies suffered from major weaknesses in design or ascertainment of diagnosis, were biased by missing data, or used insufficient statistical methods. Such faultiness might have hampered the weighted risk ratios, and so there is a need for meta-analyses based only on publications fulfilling good scientific standards. 

One of the biggest public insurance companies in the Swedish labour market (AFA Insurance) needed a scientifically based standard for evaluating work-related disease and commissioned us to perform an updated foundation for decisions regarding both the prevention and compensation of damages. We conducted a systematic review of the published epidemiological studies on work-related exposures with regard to ALS. Meta-analyses were carried out on exposures/occupations identified in three or more studies that fulfilled good scientific standards. Our first report was published in 2014 in a Swedish peer-review scrutinized series of publications https://gupea.ub.gu.se/handle/2077/37930. The aim of the present publication is to update our previous review and meta-analyses and present the results of summary measures for an international audience.

## 2. Methods

### 2.1. Literature Search

We identified relevant published articles using bibliographic search engines in PubMed, Embase, and Arbline prior to 15 February 2017. Our search criteria were the MeSH terms (Medical Subject Headings) for study design (cohort, epidemiol*, epidemiologic studies) in combination with the MeSH terms for exposure (employment, workplace, professions, career, career choice, job, occupations, employment, occupational health, occupational medicine, occupational exposure, occupational injuries, occupational diseases, electromagnetic field) in combination with any of the MeSH terms for the disease of interest (motor neuron disease, amyotrophic lateral sclerosis, primary lateral sclerosis). This search produced 372 articles. After we had scrutinized the titles and/or abstracts, excluded a few duplicates and included only articles based on original data on exposures related to occupation, 135 articles remained. The full papers were read and 79 were found to be relevant.

### 2.2. Quality Classification 

We scrutinized all relevant publications according to the checklist proposed by the MOOSE group [12]. According to the GRADE guidelines we considered selection bias and ‘falling off’ [13], as well as the occurrence of dose-response effects [14]. We also used a system for grading the degree of evidence in observational epidemiologic articles into a global class ranging from I–V, as proposed by Armon [15]. Based on these documents, we constructed a decision protocol (shown in the Appendix A) involving the categories Diagnosis, Exposure, Study Group (selection, controls, missing data), Methods and Analysis, Armon Global Score, and Funding and Exposures; see Table 1 and Table 2. The checklist by Armon and our decision protocol was also presented in a recent publication [16]. A slightly updated version is presented in the Appendix A of the present publication. The quality of the diagnosis was graded as: 1 = the El Escorial criteria [17] were applied, 2 = diagnosis from a hospital (in-patients), 3 = diagnosis from a general practitioner (GP) (also including mortality registers), and 4 = ALS was not separated from other types of muscular atrophies. The other categories were graded 1 = good, 2 = sufficient, 3 = uncertain/insufficient, or 4 = unacceptable. Sometimes a category was graded in between, and thus was given an interval, for example 2–3. The reason for this was usually lack of sufficient information for carrying out a more specific categorization.

Prerequisites for accepting a publication as fulfilling good scientific standards (Armon global score II or III) were that diagnosis had to achieve a score of 1–3 and all the other categories should be scored as 1–2 as a single score or be included in the interval 2–3. Articles not qualifying for global score II–III were impaired by serious weaknesses (Armon score IV) or should not be paid attention to (Armon score V). None of the publications fulfilled Armon global score I, which almost requires an experimental design.

Only studies [18,19,20,21,22,23,24,25,26,27,28,29,30,31,32,33,34,35,36,37,38,39,40,41,42,43,44,45,46,47,48,49,50,51,52,53,54] fulfilling good scientific standards (Armon class II or III) were used in our meta-analyses; see Table 1. Another 46 relevant publications [3,9,55,56,57,58,59,60,61,62,63,64,65,66,67,68,69,70,71,72,73,74,75,76,77,78,79,80,81,82,83,84,85,86,87,88,89,90,91,92,93,94,95,96,97,98] not fulfilling good scientific standards regarding the exposures under study are summarized in Table 2.

### 2.3. Statistical Analysis

Risk estimates from the selected studies are reported as relative risks (RR), as the outcome is rare, and so odds ratios (OR) and hazard ratios (HR) can be considered equivalent to the RR. When unadjusted as well as multivariable adjusted risk estimates were reported, we only considered the adjusted estimates. Studies which reported stratified estimates for sex were considered as separate studies and included with both estimates. When exposure was categorized into different levels, the risk rate for the highest level was used according to the principle of dose-response [99] provided a sufficient number of exposed cases was observed, usually around 30 or more. Estimates based on an extremely small number of individuals were not included, as their effect on the combined estimate would be negligible.

We examined the fixed effects model as well as the random effects model by considering statistical heterogeneity. To this end, we used the I^2^ statistic where the recommended cut-offs of 25%, 50%, and 75% degrees of heterogeneity were considered. We stratified on study characteristics (gender, source of funding, exposure characteristics, previous versus recent studies), selected a priori, and used meta-regression to evaluate the significance of the stratification variable. Based on the I^2^ criterion and the meta-regression, a random effects model was found to be the most appropriate choice in all of our analyses; hence the results are reported only with random effects estimates. The weights used for pooling the risk estimates were equal to the inverse-variance weighting. Pooled risk estimates are presented with 95% confidence intervals (CI). We also performed leave-one-out analysis for each study, to check its influence on the combined estimate as well as the degree of heterogeneity. To illustrate the progress in accumulated information about the influence of occupational exposure on ALS, we used a cumulative meta-analysis where the pooled estimate is updated each time the results of a new study have been published—a meta-cumulative approach.

Publication bias was analysed by inspection of the funnel plot, in which the estimates of RR should be distributed symmetrically around the weighted RR unless the publication was affected by bias. The rank correlation test proposed by Begg and Mazumdar [100] was used to supplement the interpretation of the funnel plot. Statistical analyses were conducted using procedures for different aspects of meta-analysis available in STATA software (version 14.2, StataCorp, College Station, TX, USA), and described in articles from the STATA journal [101].

## 3. Results

### 3.1. Exposure to Heavy Physical Activities at Work

**Figure 1:**
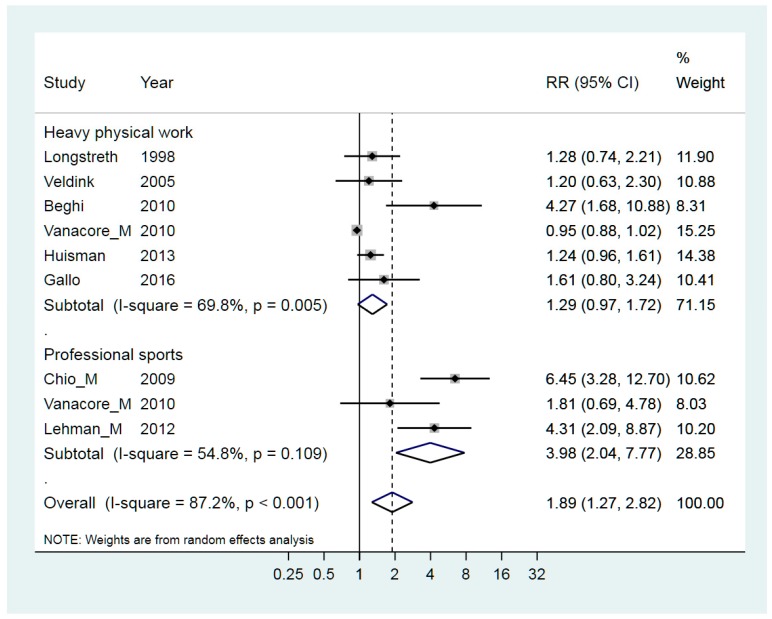
Forest plot for studies assessing the association between ALS (amyotrophic lateral sclerosis) and occupational exposure to heavy physical work. Results for men only are indicated by M; otherwise the results concern both sexes. Random effect models were used, with stratification of activities. Heterogeneity was tested by the I^2^ statistic, with *p* < 0.05 indicating rejection of homogeneity. RR = relative risk.

Since the object of our study was restricted to occupational exposures, only risk rates directly related to physical activities at work were considered. Published reviews and meta-analyses have shown that the RR is not elevated for moderate physical activity and thus we only included the results from studies on occupational exposure to heavy physical work [18,25,30,37,50,53] and likewise work as a professional athlete [20,36,50]. The weighted RRs were 1.29 (95% CI 0.97–1.72) and 3.98 (95% CI 2.04–7.77) respectively and the overall weighted RR was 1.89 (95% CI 1.27–2.82) (Figure 1). The difference in RR between the two exposure groups was significant according to the meta-regression, *p* = 0.017. There was a slight publication bias indicated in the funnel plot for heavy physical work, also shown by Begg’s test. The heterogeneity in each subgroup was lower than in the combined group and leave-one-out of Vanacore’s [50] RR for sports completely eliminated the heterogeneity for the stratum of professional sports. In the stratified analysis for gender, those studies that included both men and women had an RR of 1.45 (95% CI 1.06–2.00) and a comparably low I^2^ = 39.7%, and those with only men had an RR of 2.58 (95% CI 0.85–7.81) and a high heterogeneity I^2^ = 93.8%. However, the difference in RR was not statistically significant, *p* = 0.38.

### 3.2. Exposure to Chemicals

**Figure 2:**
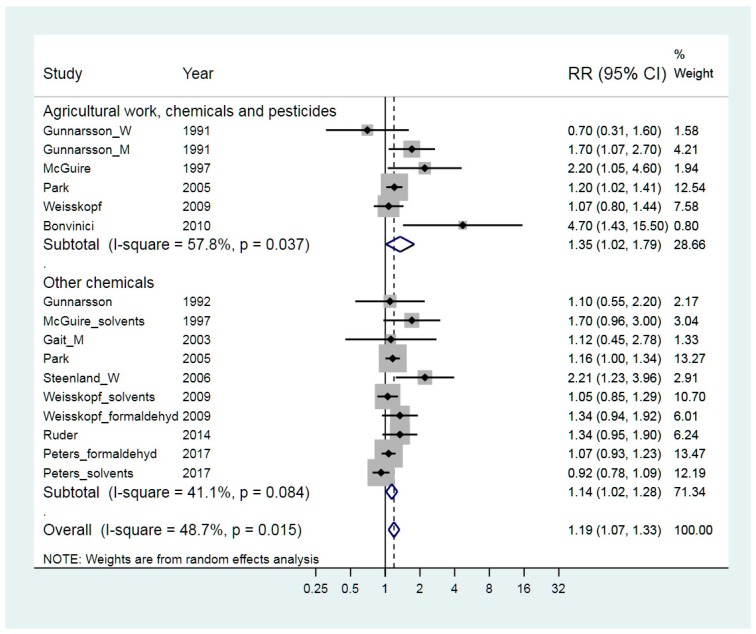
Forest plot for studies assessing the association between ALS (amyotrophic lateral sclerosis) and occupational exposure to chemicals. Results for men only are indicated by M, results for women only are indicated by W; otherwise the results concern both sexes. Random effect models were used, with stratification of exposure related to chemical exposure related to agricultural work or other chemical exposures. Heterogeneity was tested by the I^2^ statistic, with *p* < 0.05 indicating rejection of homogeneity.

The relevant studies are presented in Figure 2 and the weighted RR was slightly elevated; 1.19 (95% CI 1.07–1.33). Pesticides are designed to disturb biological systems and involve a significant risk for Parkinson’s disease [16]. Thus, we made a separate sub-analysis on the risk for ALS when occupationally exposed to agricultural work and/or pesticides, which was addressed in five publications [19,26,38,40,51]. The weighted RR was 1.35 (95% CI 1.02–1.79). Exposure to other chemicals was considered in eight studies [24,27,38,40,42,43,49,52] and the weighted RR was 1.14 (95% CI 1.02–1.28). The two exposure strata had similar heterogeneity as the combined data and their RRs were not different in the meta-regression test, *p* = 0.44.

**Figure 3:**
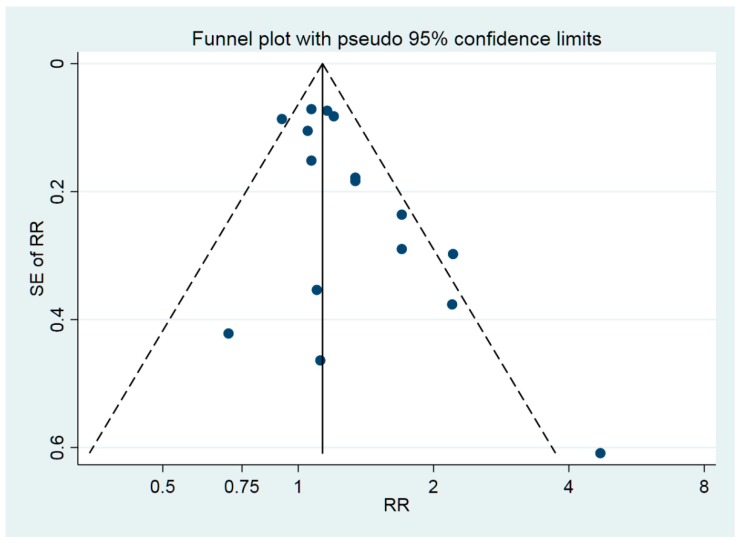
Funnel plot for the 16 RR (relative risk) estimates of the association between ALS (amyotrophic lateral sclerosis) and occupational exposure to chemicals in Figure 2.

The funnel plot (Figure 3) indicated no publication bias, supported by Begg’s test. Regarding exposure to agricultural chemicals, the heterogeneity was considerable. In the group “Other chemicals”, the heterogeneity was 41% but was reduced to 19% if the RR for solvents in the study by Peters was left out.

Stratified meta-analysis regarding funding resulted in a weighted RR of 2.11 for those three studies that had not received public funding [19,24,49] compared to an RR of 1.14 for all the other studies. This difference was statistically significant, *p* = 0.044. Other stratifications had only a small effect on RR and heterogeneity.

### 3.3. Exposure to Metals

**Figure 4:**
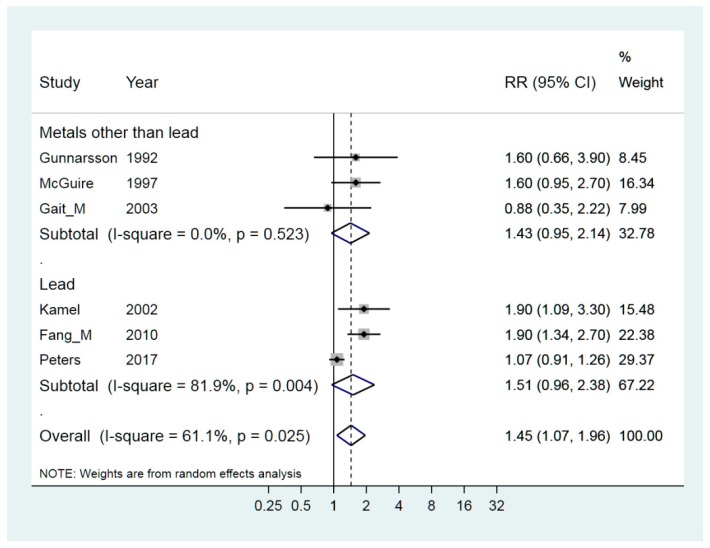
Forest plot for studies assessing the association between ALS (amyotrophic lateral sclerosis) and occupational exposure to metals. Results for men only are indicated by M; otherwise the results concern both sexes. Random effect models were used, with stratification of exposure to lead or other metals. Heterogeneity was tested by the I^2^ statistic, with *p* < 0.05 indicating rejection of homogeneity.

The weighted RR for exposure to lead [21,35,42] was 1.51 (95% CI 0.96–2.38) and exposure to metals from different sources (plants, shipyards, grinding) [24,27,38] resulted in a weighted RR of 1.43 (95% CI 0.95–2.14). The overall weighted RR was statistically significantly elevated at 1.45 (95% CI 1.07–1.96) (Figure 4). Taking into consideration that there were only six studies, there was an evident publication bias seen in the funnel plots and also by Begg’s test. The leave-one-out analysis showed that the heterogeneity vanished if the studies by Peters and Gait respectively were left out. The analysis of strata showed that heterogeneity was not present for the exposure to other metals, I^2^ = 0.0%; and was low for non-public funding, I^2^ = 17.7%, but the number of studies is very small in this stratified analysis.

**Figure 5:**
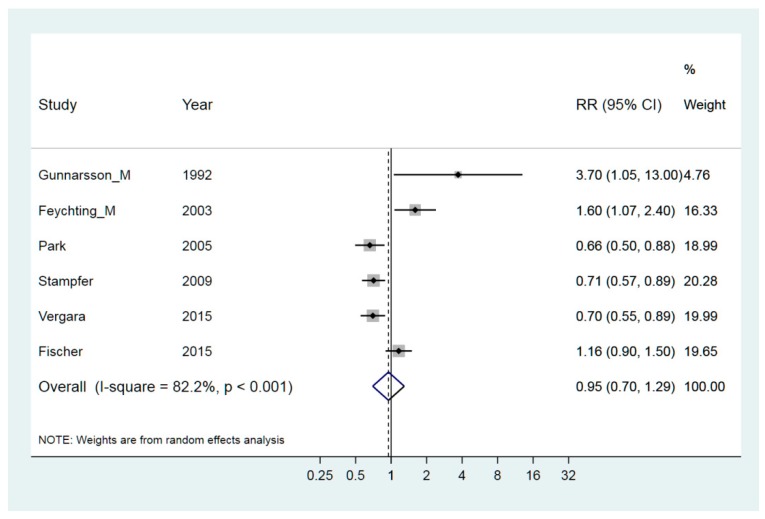
Forest plot for studies assessing the association between ALS (amyotrophic lateral sclerosis) and occupational exposure to welding. Results for men only are indicated by M; otherwise the results concern both sexes. Random effect models were used. Heterogeneity was tested by the I^2^ statistic, with *p* < 0.05 indicating rejection of homogeneity.

Welding involves both exposure to fumes of vaporized metals and high electromagnetic fields. Focusing only on the first aspect (vaporized metals), we used risk rates based on years at work with welding and the weighted RR was 0.95 (95% CI 0.70–1.29), (Figure 5) [22,23,27,32,40,48,51,54]. The heterogeneity was apparent and leaving out the big study by Feychting [22], it was reduced from 82% to 77%.

### 3.4. Exposure to Electromagnetic Fields and Work with Electricity

**Figure 6:**
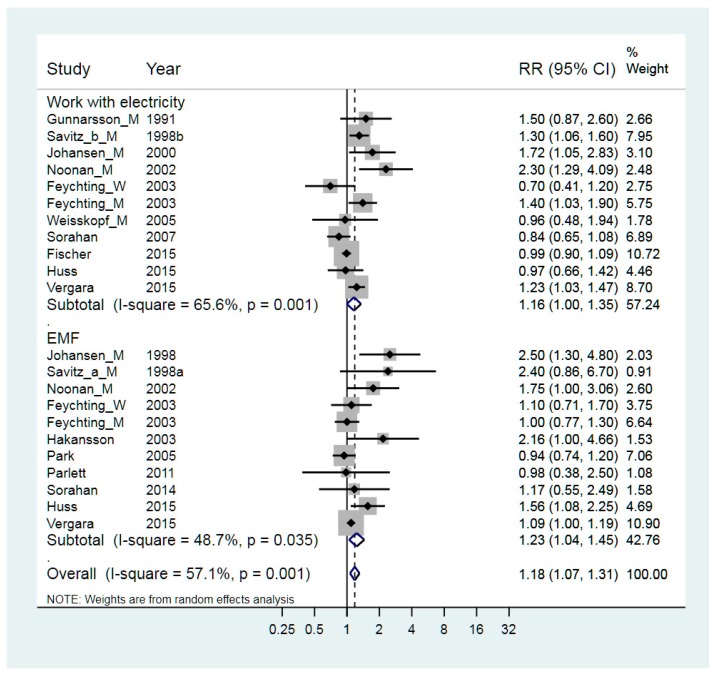
Forest plot for studies assessing the association between ALS (amyotrophic lateral sclerosis) and occupational exposure to work with electricity and electromagnetic fields (EMF). Results for men only are indicated by M, results for women only are indicated by W; otherwise the results concern both sexes. Random effect models were used, with stratification of exposure to work with electricity or EMF. Heterogeneity was tested by the I^2^ statistic, with *p* < 0.05 indicating rejection of homogeneity.

Work with electricity in relation to ALS has been extensively studied (Table 1). The exposures can be separated into electrical accidents/trauma and electromagnetic fields (EMF) per se. The level of EMF exposure was estimated through a job exposure matrix (JEM) established by occupational hygienists [22,31,32,33,39,40,41,44,47,54], and the weighted RR was 1.23 (95% CI 1.04–1.45), which is statistically significant (Figure 6).

When the focus is on electrical accidents/trauma, the exposure is based on job titles and years of work with electricity [22,23,26,31,34,39,45,46,51,54], resulting in the slightly elevated weighted RR = 1.16 (95% CI 1.00–1.35). Meta-regression showed that there was no statistically significant difference between the two types of exposure, *p* = 0.57, and the heterogeneity was almost the same. Stratification on gender gave RR = 1.38 for studies with men only, RR = 1.10 for both men and women and RR = 0.90 for women only. The difference in RR for these three groups was, however, not statistically significant, *p* = 0.74. The heterogeneity was substantially lower for studies with men only and with women only (I^2^ = 36.0% and 38.8% respectively).

A slight publication bias indicated by the funnel plot was supported by Begg’s test. The leave-one-out test showed that the RR for EMF differed between 1.16 and 1.32 and for occupations working with electricity between 1.12 and 1.21, and had thus no significant impact on the results. The highest impact on heterogeneity was the exclusion of the study by Johansen [33], where heterogeneity for studies with EMF decreased from 48.7% to 33.5%, but the impact on the value of RR was more modest, from 1.23 to 1.16.

**Figure 7:**
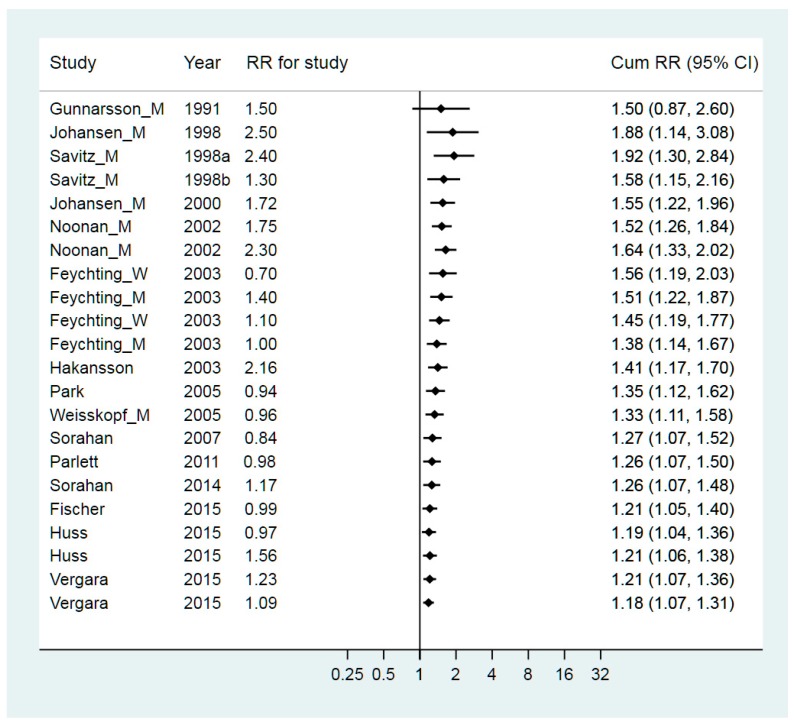
Cumulative meta-analyses on the association between ALS (amyotrophic lateral sclerosis) and occupational exposure to EMF or work with electricity with the pooled estimate (Cum RR) updated for every new study, year by year.

A meta-analysis over the time period 1991–2015 in which the results of earlier studies were gradually cumulated showed a successive reduction of RR (Figure 7). For the period 1991–2005, the weighted RR was 1.33 (95% CI 1.11–1.58), compared to the period 2006–2015 with a weighted RR of 1.18 (95% CI 1.07–1.31).

### 3.5. Nursing and Medical Treatment

Previous literature reviews have not indicated that exposure to medical work might involve increased risk for ALS. However, in four publications, the risk posed by working as a nurse or physician has been addressed [26,40,42,51]. The heterogeneity was low and thus the weighted risk estimate was based on the fixed model resulting in an RR of 1.18 (95% CI 1.05–1.34).

## 4. Discussion

Meta-analyses based only on epidemiologic publications of good scientific quality showed that the risk of amyotrophic lateral sclerosis was statistically significantly elevated for occupational exposures to heavy physical work, metals (especially lead) and chemicals (especially pesticides).

In the old study by Breland [3], elevated risk of ALS was stated for heavy work load involving the occupations farmers/farmworkers, construction workers and professional athletes. Our results support that these groups have a higher risk of being afflicted by ALS.

The risk for ALS in professional athletes was four times higher than that of the reference population, while high work load in other occupations only involved on average a 30% increased risk. The Italian study [20] included 7000 professional soccer players together with 1900 basketball players and 1700 cyclists. The mortality from ALS was not elevated among the latter two groups of athletes. The possibility that other exposures (for example chemicals) related to playing soccer may also be risk factors for ALS is also discussed. Since our meta-analysis showed that exposure to pesticides or agricultural chemicals resulted in a 35% increased risk, one mechanism might be that a combination of sports-related muscle trauma and pesticides associated with the pitch contribute to the four-fold greater risk for ALS.

A recent case-control study (175 patients and 317 controls) of good scientific quality using a validated questionnaire for historical physical activity showed that casual exercise-related physical activity was associated with an elevated risk of ALS (adjusted RR 1.28 with 95% CI 1.06–1.55), while work-related physical activity did not involve an increased risk (adjusted RR 1.02 with 95% CI 0.97–1.08) [28]. That study did not present risk rates for occupationally heavy physical work and could thus not be included in our meta-analyses. However, the risk rate for casual exercise almost equalled the weighted RR for heavy occupational physical work in our meta-analysis.

In a study among 2.5 million military personnel, 107 cases of ALS were identified. Those who were deployed to the Gulf Region during the Gulf War had an almost two-fold greater risk of ALS compared to those who did not participate in the Gulf War [102]. Risk factors discussed are both physical trauma and chemicals. Confounders such as head trauma (in the case of Gulf veterans and professional athletes) and increased metabolism [103] caused by heavy physical work and agricultural work also might have an impact on the risk for ALS.

Construction workers are exposed to both muscle trauma and metals. The latter exposure involved an almost 50% increased risk according to our meta-analysis. Exposure to lead was assessed in three studies, of which two fulfilled the best scientific standards (global class II) where the diagnosis was assessed by a neurologist [21,35]. One study was based on ALS patients and information on exposure was gathered from a questionnaire [35], and the other study only involved US veterans exposed to lead from firing practice [21]. In both studies, the patients afflicted with ALS also had elevated levels of lead in their blood compared to controls. The third study (global score III) was based on hospital registers for patients with ALS compared to population controls [42]. Exposure was estimated from the Swedish census and a job exposure matrix; thus, the quality of both the diagnosis and exposure was less valid than in the other two studies. To sum up, the RR of 1.90 for lead exposure from the first two studies is considerably more valid than the RR of 1.07 from the last study.

In comparison, there is a study of the levels of lead in the blood of patients with ALS in an Italian agricultural area [104]. This study was not included in our meta-analysis since data on exposure were missing. However, the patients had significantly higher blood levels of lead than population controls.

Occupational exposure to pesticides involved a statistically significant elevated risk (RR 1.35). The highest impact on the RR was shown in the Italian study by Bovinci [19] involving 41 cases and 82 controls. Although this study was funded by a patient organization, the study design was scientifically appropriate and fulfilled the criteria for Armon class II. Thus, in this case, the lack of public funding did not bias the study.

During the turn of the millennium, some studies indicated a doubled risk of ALS after exposure to electromagnetic fields or work with electricity. This stimulated further studies of different design and the cumulative risk rate has gradually diminished (Figure 7), probably because publication bias was more common before 2005. Possibly harmful exposures might be associated with EMF, such as electric shocks causing muscle trauma. In selecting appropriate risk rates from the publications, we tried to differentiate between exposures to EMF or work with electricity, but the weighted risk rates were almost identical. Welding involves exposures to strong electromagnetic fields and welders are included in the job exposure matrices used to estimate exposure to EMF. However, a separate meta-analysis of welders showed no risk (RR = 0.95), which might indicate that exposures other than EMF might involve risk for ALS in this population.

Some studies indicate a 50% increased risk of ALS among health care personnel [26,40,51]. A nation-wide nested case-control study [42] of ALS in Sweden from 1991–2010 resulted in an RR of 1.13 (95% CI 0.99–1.29) and the weighted cumulated RR over time is only slightly elevated.

General limitations of meta-analyses are that the calculations can only be based on published data and will reflect any inherent weaknesses of design in the studies included. Furthermore, all previously published meta-analyses on ALS have been based on all relevant publications identified, irrespective of the quality of the study design.

One strength of our study is that our meta-analyses were based on a systematic literature review, only including studies that fulfilled scientifically high-quality standards. Based on the detailed checklist proposed by Armon [15], we used an elaborated protocol for scrutinizing publications [16]. Another strength of our meta-analyses is that we focused heavily on finding all possible sources of bias, using stratification of data with regard to possible confounders such as gender and funding. We also looked for publication bias using both funnel plots and a test for publication bias.

In 25 out of the 37 studies, the diagnosis of ALS was made by a general practitioner or a mortality register [20,22,23,24,25,26,28,31,32,34,36,39,40,41,42,43,44,45,46,48,49,50,51,52,54]. In these studies, true cases of ALS (fulfilling the common diagnostic criteria) might have been mixed with a few cases of motor neuron disease mimics. However, such non-differential misdiagnoses are not expected to bias the risk estimates, but only cause wider confidence intervals.

Regarding exposure [15], we made sure that only exposures occurring a few years before the start of overt disease were considered and that methods of gathering information on exposure were the same for both affected and healthy individuals. In some studies, exposure was assessed by occupational hygienists with a job exposure matrix or established by a specifically exposed cohort. In the case of questionnaires/interviews, it is also more likely that occupational hygienists were involved (it was not always clear from the text). The heterogeneity of results between studies could be explained by the use of different measures regarding exposure, comparing results from JEM with questionnaires/interviews or exposed cohorts. The leave-one-out analyses verified that this was one of the main reasons for heterogeneity. Another factor that could lead to increased heterogeneity is the variation in geography, that is, nations where the exposure took place. Working conditions are certainly not the same in different countries, but the data at our disposal is too sparse to analyse this in depth. Several study groups were hampered by bias caused by missing data (low response rate) from cases, controls or inappropriate matching of controls. Regarding methods and analysis, attention was paid to sources of bias and confounding as well as the use of appropriate statistical methods. Based on all these parameters, both authors individually examined all relevant publications using a structured template summarizing different aspects of design, exposure, methods and results. The results of our examinations were condensed into the graded scores in Table 1. If our scores were divergent, we re-examined the publication and, after discussion, reached consensus. After a ‘run-in period’, we got used to the protocol for grading different aspects of studies and the consensus between us became almost complete; there were only very few cases where a consensus discussion was necessary. However, there is always room for a reader’s own discretion when judging a publication.

## 5. Conclusions

Our meta-analyses suggest positive associations between ALS and occupational exposures to excessive physical work, pesticides and possibly also to electromagnetic fields and health care work, and the results are not explained by publication bias. A positive association was also found for metals, but the few available studies indicated some degree of publication bias, making the results somewhat less valid. Further epidemiological studies should also consider confounders such as head trauma and increased metabolism, which is associated with heavy physical work and agricultural work.

## Figures and Tables

**Table 1:** Publications fulfilling good scientific standards (Armon class II or III) [15] according to the algorithm presented in the Appendix A.

Publication	Year	Diagnosis	Exposure		Study Group: Selection, Controls, Missing Data		Methods Analysis	Armon Global Class [15]	Funding:	Exposures
			Assessment	Grade	Number of Exposed Cases	Grade				
Beghi [18]	2010	1	Q	2	31	2–3	2–3	III	PU	Hard physical work, physical trauma
Bonvicini [19]	2010	1	Q	2	13	2	2	II	PA	Chemicals (pesticides)
Chio [20]	2009	2–3	EC	2	8	2	2–3	III	PA, PU	Professional sports
Fang [21]	2010	1	EC	2	151	1	1	II	PA, PU	Lead
Feychting [22]	2003	3	JEM	2–3	234 *	1	1	III	PU	Occupation (work with electricity, EMF, welding)
Fischer [23]	2015	2–3	JEM	2	766 *	1	1	III	PU, I	Occupation (EMF, welding)
Gait [24]	2003	3	JEM	2	13 *	2–3	2	III	?	Metals and solvents
Gallo [25]	2016	3	Q	2–3	17	2	2–3	III	?	Hard physical work
Gunnarsson [26]	1991	3	JEM	2	32 *	2	2	III	PU	Occupation (electricians, chemicals, nurse)
Gunnarsson [27]	1992	1	Q	2	10 *	2	1	II	PU	Metals, chemicals, welding
Harwood [28]	2016	2	Q	2	175	2	2	III	PU	Work related physical activity
Horner [29]	2008	1	EC	2	48	2	2	II	PU	Gulf War veterans
Huisman [30]	2013	1	Q	1	103	2	1	II	PU	Hard physical work
Huss [31]	2015	3	JEM	2	46 *	2	1	III	PU	Occupation (work with electricity, EMF)
Håkansson [32]	2003	3	JEM	2–3	13	2	1	III	I	Occupation (EMF)
Johansen [33]	1998	3	JEM	2–3	14	2	2	III	PU, I	Occupation (EMF)
Johansen [34]	2000	2	JEM	2	15	2	2	II	PU, I	Occupation (work with electricity)
Kamel [35]	2002	1	Q	1	11	2	1	II	PU	Lead
Lehman [36]	2012	3	EC	1	7	2	2	III	PU	Professional sports
Longstreth [37]	1998	1	Q	2	59	2–3	2	III	PA	Hard physical work
McGuire [38]	1997	1	Q	2	71	2–3	2	III	PU	Chemicals, metals
Noonan [39]	2002	3	JEM	2–3	19 *	2	1–2	III	?	Occupation (work with electricity, EMF)
Park [40]	2005	3	JEM	2–3	5965 *	2	2	III	PU	Occupation (EMF, pesticides and other chemicals, welding, nurse)
Parlett [41]	2011	3	JEM	1	10	2–3	2–3	III	none	Occupation (EMF)
Peters [42]	2017	2–3	JEM	2–3	611 *	1	1	III	PA, PU	Occupation (chemicals, lead, nurse)
Ruder [43]	2014	3	EC	1	20	2	2	III	PU	Chemicals (PCB)
Savitz [44]	1998a	3	JEM	2–3	9	2	2	III	I	Occupation (EMF)
Savitz [45]	1998b	3	JEM	2–3	114	2	2	III	?	Occupation (work with electricity)
Sorahan [46]	2007	3	JEM	2	62	2	2	III	I	Power station workers (work with electricity)
Sorahan [47]	2014	3	JEM	2	11	2	2	III	I	Power station workers (EMF)
Stampfer [48]	2009	3	JEM	2–3	71	2	2	III	I	Occupation (welding)
Steenland [49]	2006	3	JEM	1	11	2	2	III	?	Chemicals (PCB)
Vanacore [50]	2010	3	JEM	2–3	6	2–3	2–3	III	?	Hard physical work, professional sports
Weisskopf [51]	2005	3	Q	2–3	30 *	2	2	III	?	Occupation (work with electricity, nurse)
Weisskopf [52]	2009	3	Q	2–3	142	2	2	III	PU	Chemicals
Veldink [53]	2005	1	Q	2	25	2–3	2–3	III	PU	Strenuous physical work
Vergara [54]	2015	3	JEM	2–3	503 *	2–3	1–2	III	PU	Occupation (EMF, work with electricity, welding)

Diagnosis was graded with a score from 1–3 (diagnosis from a neurologist, in-patient care or general practitioner (GP)/mortality register). The other categories were graded with either a single score of 2 or were included in the interval 2–3 (1 = good, 2 = sufficient, 3 = uncertain/insufficient). Assessment of exposure: JEM = Job Exposure Matrix, Q = questionnaires/interviews, EC = exposed cohort. EMF = electromagnetic fields, PCB = polychlorinated biphenyls. Funding: I = industry, PA = patient association, PU = public, ? = funding is not possible to classify based on information in source text. * In case of multiple exposures, the highest number of exposed cases is registered.

**Table 2:** Publications not fulfilling good scientific standards [3,9,55,56,57,58,59,60,61,62,63,64,65,66,67,68,69,70,71,72,73,74,75,76,77,78,79,80,81,82,83,84,85,86,87,88,89,90,91,92,93,94,95,96,97,98] (Armon class IV or V) [15] according to the algorithm presented in the Appendix A.

Publication	Year	Diagnosis	Exposure	Study Group: Selection Controls Missing Data	Methods Analysis	Armon Global Class [15]	Funding	Exposures
Andrew [55]	2017	3	3–4	4	2–3	V	PU, PA	Chemicals, metals, water-related activities
Armon [56]	1991	1	2	3	2–3	IV	PU	Physical work, trauma, metals
Belli [57]	2005	3	2	2	3	IV	?	Professional soccer player
Bertke [58]	2016	3	3	2	3–4	IV	PU	Metals
Binazzi [59]	2009	1	2	3	2–3	IV	?	All possible
Breland [3]	1967	1–2	3	2–3	3	IV	?	Physical work
Buckley [60]	1983	3–4	3	2–3	3	IV	PU	Occupation
Burns [61]	2001	3	1	2	3	IV	I	2,4-dichlorophenoxyacetic acid
Chancellor [62]	1993	1	2	3	2–3	IV	PA	Chemicals, manual work
Chiò [63]	1991	1	3	3–4	3	IV	I	Occupation
Das [64]	2012	1	3	3	3	IV	PU	Pesticides
Davanipour [65]	1997	1	2	3	2–3	IV	?	EMF
Deapen [66]	1986	2–3	2	3	3	IV	PA	Physical trauma (electric shock)
Fang [67]	2009	1	2	3	2–3	IV	PU	Chemicals
Furby [68]	2010	1	2	3	2–3	IV	I	Agricultural work and chemicals
Gallagher [69]	1987	1	2	4	4	V	?	Physical trauma
Govoni [70]	2005	1	4	3	3	V	PU	Living in an agricultural area
Graham [71]	1997	3	2–3	3	2–3	IV	PU	Chemicals
Granieri [72]	1988	1	2	3	2–3	IV	PU	Occupation (Physical work, farmer, forest work)
Gresham [73]	1986	1	2–3	3	3	IV	?	Metals
Gunnarsson [74]	1996	1	4	2	2–3	V	PU	Living in an agricultural area
Kalfakis [75]	1991	1	3	4	4	V	?	Occupation
Kamel [9]	2012	1	2	3	2–3	IV	PU	Pesticides
Kondo [76]	1981	3	2–3	3	3	IV	PU	Physical trauma
Kurtzke [77]	1980	3	3	2	2–3	IV	PU	All possible
Lewis [78]	2000	3	3–4	2	2	IV	I	Work at a petroleum company
Malek [79]	2014	1	2–3	3–4	2–3	IV	PA	Metals, pesticides
Mitchell [80]	1995	1	2	3	3	IV	PU	All possible
Morahan [81]	2006	1	2–3	3–4	3	IV	PA	Solvents, pesticides
Pamphlett [82]	2012	1	2–3	3–4	3	IV	PA	Solvents, pesticides (extension of Morahan 2006)
Pamhplett [83]	2013	2	3	4	3	V	PA	Diesel
Pinkerton [84]	2013	3	3–4	2	3	IV	PU	Chemicals (formaldehyde)
Pinkerton [85]	2016	3	3–4	2	3–4	IV	PU	Flight attendant
Provinciali [86]	1990	1	3	3–4	3	IV	?	Metals, trauma, heavy manual labour
Pupillo [87]	2014	1	2	3	1–2	IV	PU, I	Physical work
Roberts [88]	2015	3	3	2–3	3	IV	PA	Formaldehyde
Roelofs-Iverson [89]	1984	1	2–3	4	3	V	PU	Heavy metals
Röösli [90]	2007	3	1	2	3	IV	PU	Occupation (EMF)
Savettieri [91]	1991	3	2–3	4	2	V	PU	All possible
Scarmeas [92]	2002	1	2	3	2–3	IV	?	Athletic activity
Schulte [93]	1996	3	3	2	3	IV	?	Occupation (farmers)
Strickland [94]	1996	1	2–3	3	2–3	IV	PU	Metals
Strickland [95]	1996	1	2–3	3	2–3	IV	PU	Physical work
Sutedja [96]	2007	1	2–3	3	2	IV	PU	Occupation (education)
Valenti [97]	2005	1	3	3	3	IV	?	Athletic activity
Yu [98]	2014	1	1–2	3–4	3	IV	PU, I	Physical activity, metals, pesticides

These publications have either Diagnosis graded as 4 or any other category (Exposure, Study Group, Methods) graded with a single score of 3 or 4 (3 = uncertain/insufficient, 4 = unacceptable). I = industry, PA = patient association, PU = public, ? = funding is not possible to classify based on information in source text, EMF = electromagnetic fields.

## Appendix A

### Appendix A.1. Decision Protocol

A modified system for categorisation of observational epidemiologic articles into classes I–V (based on Armon [15]) was applied for cohort studies with parallel controls and case-control studies, respectively. This appendix is based on a recent publication [16] and has been further adjusted.

### Appendix A.2. Diagnosis

Cohort studies:

- Diagnosis of the studied disease made by applying uniform efforts and criteria to exposed and unexposed cohorts.

Case-control studies:

- Demonstration that ascertainment of patients is complete in the given population.
- Diagnosis of the studied disease made by applying established criteria.

### Appendix A.3. Exposure

Cohort studies:

- Exposure, and hence assignment to the ‘exposed’ cohort, established before knowledge of diagnostic status, or without knowledge of diagnostic status, or confirmable independently of the knowledge of diagnostic status. Consideration of and accounting for possible misclassification.
- Unexposed cohort is appropriate to the risk factor in question, is well-matched to the exposed cohort on factors other than the exposure, and is otherwise representative of the general population.
- Exposure quantified, where possible, to permit assessment of dose-response relationships.

Case-control studies:

- Putative risk factor or exposure occurred before probable biologic onset of disease.
- Uniform effort to gather information equally from affected and unaffected individuals.
- Blinding of the information-gathering method to individuals’ disease status ideal; if not done—adequate justification as to why this does not affect the assessment of the risk factor in question.
- Blinding of subjects and individuals gathering the data as to the hypotheses being tested. If not done—adequate justification as to why this does not affect the assessment of the risk factor in question.
- Meticulous attention to avoiding recall bias or, if not possible, to evaluating its impact, estimating the magnitude of its impact and controlling for it.
- Exposure quantified, where possible, to permit assessment of dose-response.

### Appendix A.4. Study Group

Cohort studies:

- Loss to follow-up low, and comparable in exposed and unexposed cohorts. Possible roles of competing causes of mortality accounted for. Preferably, all mortality data available for both cohorts.

Case-control studies:

- Appropriate choice of controls to assure they are matched to the patients and are also representative of the general population (assure adequate matching, avoid ‘overmatching’).
- High response rates from patients and controls.

### Appendix A.5. Methods and Analysis

Cohort studies:

- Sources of biases and confounding identified and accounted for.
- Conclusion based on large numbers. Appropriate statistical analysis.

Case-control studies:

- Sources of biases and confounding identified and accounted for.
- Conclusions based on large numbers. Appropriate statistical analysis. Methods state if hypotheses were selected a priori for confirmatory analysis.

### Appendix A.6. Criteria for Categorization into Armon Class

In order to form the basis of the Armon classes I–V, we constructed a protocol with standardised criteria for grading the categories Diagnosis, Exposure, Study Group and Methods and Analysis. The protocol was graded into scores of 1 to 4 as defined below. Because of a lack of information, sometimes a category had to be graded in between, and thus given an interval, for example 2–3.

Diagnosis: 1 = the specific disease diagnosis was established by a specialist, 2 = diagnosis obtained from a hospital register (in-patients), 3 = diagnosis from a GP (also including mortality registers), and 4 = the specific disease diagnosis was not separated from other diagnoses with similar phenotype.

Exposure, Study Group and Methods and Analysis: 1 = good, 2 = sufficient, 3 = uncertain/insufficient, or 4 = unacceptable.

- Class I: All criteria were met within the respective design, i.e., a single score of 1 in every category.
- Class II: Single score of 1 or 2 in every category.
- Class III: Single score of 3 allowed for Diagnosis and 1 or 2 in the other categories (interval 2–3 is allowed).
- Class IV: Single score of 3 in categories other than Diagnosis.
- Class V: Single score of 4 in any category.
